# Adjuvant treatment of early-stage melanoma by local i.d. administration of low-dose CpG-B and GM-CSF increases recurrence-free survival: long-term follow-up of three randomized clinical trials

**DOI:** 10.1186/2051-1426-3-S2-P201

**Published:** 2015-11-04

**Authors:** Tanja D de Gruijl, Bas D Koster, Mari FCM van den Hout, Berbel JR Sluijter, Barbara G Molenkamp, Ronald JCLM Vuylsteke, Paul AM van Leeuwen, Rik J Scheper, Petrousjka van den Tol, Alfons van den Eertwegh

**Affiliations:** 1 VU University medical center, Cancer Center Amsterdam, Amsterdam, Netherlands; 2 Spaarne Gasthuis, Haarlem, Netherlands

## 

Currently, there is no adjuvant treatment available that has been shown to reduce the chances of recurrence after surgical excision of localized melanoma. Between 2002 and 2007 we conducted three randomized and placebo (saline) controlled Phase II clinical trials in which we treated clinically stage I-II melanoma patients with 1) GM-CSF (3 μg/kg), 2) CpG-B (PF-3512676/CpG7909, 8 mg), and 3) CpG-B, alone or combined with GM-CSF (2 mg and 100 μg, respectively), through 1-4 intradermal injections, directly adjacent to the primary melanoma excision scar, within a week of re-excision and sentinel lymph node (SLN) biopsy[1-3]. The trials were IRB-approved and written informed consent was obtained from all participating patients. Injections were well tolerated with only transient and mild flu-like symptoms and mild-to-moderate fevers after administration. Primary endpoint of the trials was biological efficacy; immune monitoring revealed recruitment and activation of dendritic cell subsets in the SLN as well as increased intra-SLN and systemic rates of melanoma antigen reactive CD8^+^ T cells. Here, we present clinical follow-up of all patients (n=62) who participated in these trials.

Upon pathological examination of the SLN, a remarkable difference in numbers of pathologically stage III patients was revealed, with significantly less tumor positive SLN in the CpG-B/GM-CSF administered groups (3/36 versus 9/26 in the saline control arms, p=0.020), suggesting a rapidly induced antitumor response. Follow-up further provided evidence of systemic tumor protection: in the CpG/GM-CSF-treated groups only two recurrences occurred, both within 26 months of SLN biopsy, whereas nine recurrences were observed in the saline group (range 5-113 months). In post-hoc analysis, at a median follow-up of 79 months, this translated into a significantly longer recurrence-free survival (RFS) in the CpG-treated group (p=0.010). Even for patients with pathologically confirmed and prognostically favorable stage I-II, this difference held true (p=0.017). Disease-specific survival rates at this time were 76.9% in the saline vs. 94.5% in the CpG-B/GM-CSF group.

We conclude that intradermally administered CpG-B/GM-CSF is safe and may prolong RFS. Consistent with findings from immune monitoring, apparent down-staging and prolonged RFS may result from the respective boosting of local and systemic antitumor immunity through local conditioning of the SLN. These exciting findings warrant further clinical exploration of local non-toxic immune potentiation in early-stage melanoma to prevent disease recurrence and metastatic spread.
